# Intravenous Administration of Lycopene, a Tomato Extract, Protects against Myocardial Ischemia-Reperfusion Injury

**DOI:** 10.3390/nu8030138

**Published:** 2016-03-03

**Authors:** Chao Tong, Chuan Peng, Lianlian Wang, Li Zhang, Xiaotao Yang, Ping Xu, Jinjin Li, Thibaut Delplancke, Hua Zhang, Hongbo Qi

**Affiliations:** 1 Department of Obstetrics, The First Affiliated Hospital of Chongqing Medical University, 1 Youyi Road, Yuzhong District, Chongqing 400016, China; chaotongcqmu@163.com (C.T.); thibautdelplancke@gmail.com (T.D.); 2 Canada-China-New Zealand Joint Laboratory of Maternal and Fetal Medicine, Chongqing Medical University, Chongqing 400016, China; llian_w@163.com (L.W.); Morcherl@163.com (L.Z.); xiaotaocqmu@163.com (X.Y.); aaa.abing@foxmail.com (P.X.); shinydl@163.com (J.L.); 3 Chemical Biology Research Center, College of Pharmaceutical Sciences, Wenzhou Medical University, Wenzhou 325035, Zhejiang, China; 4 Laboratory of Lipid & Glucose Metabolism, The First Affiliated Hospital of Chongqing Medical University, Chongqing 400016, China; pengchuan2096@sina.com

**Keywords:** lycopene, ischemia-reperfusion injury, myocardial infarction, ROS, inflammation

## Abstract

Background: Oral uptake of lycopene has been shown to be beneficial for preventing myocardial ischemia-reperfusion (I/R) injury. However, the strong first-pass metabolism of lycopene influences its bioavailability and impedes its clinic application. In this study, we determined an intravenous (IV) administration dose of lycopene protects against myocardial infarction (MI) in a mouse model, and investigated the effects of acute lycopene administration on reactive oxygen species (ROS) production and related signaling pathways during myocardial I/R. Methods: In this study, we established both *in vitro* hypoxia/reoxygenation (H/R) cell model and *in vivo* regional myocardial I/R mouse model by ligating left anterior artery descending. TTC dual staining was used to assess I/R induced MI in the absence and presence of acute lycopene administration via tail vein injection. Results: Lycopene treatment (1 μM) before reoxygenation significantly reduced cardiomyocyte death induced by H/R. Intravenous administration of lycopene to achieve 1 μM concentration in circulating blood significantly suppressed MI, ROS production, and JNK phosphorylation in the cardiac tissue of mice during *in vivo* regional I/R. Conclusion: Elevating circulating lycopene to 1 μM via IV injection protects against myocardial I/R injury through inhibition of ROS accumulation and consequent inflammation in mice.

## 1. Introduction

Ischemic heart diseases (IHD) are caused by insufficient blood and oxygen supply to the myocardium as a result of an arteriolar blockage. Angioplasty, coronary bypass surgery, and thrombolytic treatment are commonly used to treat patients with severe IHD. However, reperfusion after a period of prolonged ischemia can often cause myocardial ischemia-reperfusion (I/R) injury, which leads to an increase in infarct size and consequent life-threatening arrhythmias [1]. Therefore, myocardial I/R becomes a major limitation of some clinical interventional therapies for coronary artery disease.

The mechanism underlying cardiac I/R injury remains unclear, but it is reported that I/R significantly increases the production of reactive oxygen species (ROS) generated from complexes I and III of the electron transport chain (ETC) in mitochondria [2]. In addition, treatment with antioxidants such as superoxide dismutase (SOD), catalase (CAT) [3], and antioxidation inducer [4] resulted in the reduction of myocardial I/R damage. This indicates that natural potent antioxidant characteristics could be beneficial for limiting myocardial I/R injury in IHD patients by scavenging ROS.

**Figure 1:**
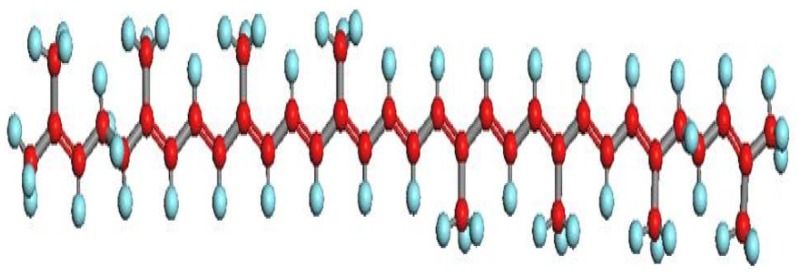
Structure of lycopene.

Lycopene is a lipophilic natural compound, and has been well studied due to its strong antioxidant properties [5]. It is now known that lycopene is a carotenoid with an open-polyene chain structure similar to β-carotene but lacking a β-ionone ring (Figure 1). It was originally isolated from black bryony, but commonly enriched from fruits and vegetables such as tomato, pepper, and papaya [6]; thus, lycopene can be easily uptaken from food resources. There are numerous health benefits associated with consuming lycopene. For example, lycopene has been shown to impede prostate cancer progression in patients with benign prostate hyperplasia [7] and protecting against amyloid β-induced neurotoxicity [8]. In addition, food supplementation of lycopene significantly decreases low-density lipoprotein (LDL) cholesterol serum levels [9] while inhibiting proinflammatory cytokine secretion in obese females [10]. Lycopene deficiency is associated with several chronic diseases including cancer [11], pancreatitis [12], gastritis [13] and atherosclerosis [14]. Notably, there is increasing evidence suggests that low levels of circulating lycopene is associated with increased risk of heart diseases [14,15].

Epidemiological studies in Europe suggested that higher lycopene concentrations in adipose tissue protect against myocardial infarction (MI) [16], while other groups have shown that prolonged exposure to lycopene attenuated myocardial I/R injury in rat model [17,18]. However, both human and animal studies have demonstrated that lycopene is easily absorbed by the body, but predominantly deposited into liver, adrenal, lungs, prostate glands and skin after processing [19,20]. This distribution pattern of lycopene may significantly compromise its benefits for heart diseases [21]; therefore, debate still remains that oral administration of lycopene is efficient enough to protect against myocardial I/R injury.

In the present study, we aimed to examine whether intravenous (IV) lycopene administration is beneficial for cardiac I/R injury, and further explore the mechanisms underlying lycopene’s effects on myocardial I/R. We attempt to provide evidence that lycopene can be acutely used to treat ischemic heart diseases.

## 2. Methods and Materials

### 2.1. Animals, Cell Lines and Chemicals

Male 10–12 weeks old C57BL/6 mice were animals were obtained from the Experimental Animal Center of Chongqing Medical University. Mice were maintained on a 12-h light–dark cycle in a controlled environment with water *ad libitum*. All animal experiments were performed in accordance with National Institute of Health (NIH) Guide for the Care and Use of Laboratory Animals. The animal experiments were approved by the Medical Ethics Committee of Chongqing Medical University. HL-1 cardiomyocytes were generously provided by Claycomb, and cultured as described previously [22]. Lycopene from tomato was purchased from Sigma (St. Louis, MO, USA).

### 2.2. In Vitro Hypoxia Model and Trypan Blue Exclusion Assay

HL-1 cells were seeded in a 12-well plate one day prior to conducting experiments. On the day of experiment, cells were at least 80% confluent and fresh supplemented Claycomb medium was applied. For hypoxia treatment groups, cells were placed in 95% nitrogen and 5% carbon dioxide in a mixed-gas perfusion hypoxia chamber. Cells were allowed to incubate for 2 h in hypoxic conditions after which solutions of varies lycopene concentrations were added and placed in reoxygenating conditions at 37 °C. Control groups were incubated in normoxia conditions at 37 °C in a cell culture incubator for 2 h followed by another 2 h incubation with vehicle in normoxia. All groups were trypsinized and resuspended in 1.5 mL HBSS buffer, and then 1:1 diluted with 0.4% trypan blue solution. After 10 min of incubation at 37 °C, cells were transferred onto a hemocytometer and counted under a microscope.

### 2.3. In Vivo Myocardial I/R Model

Mice were anaesthetized by sodium pentobarbital (50 mg/kg body weight) then placed on a ventilator (Harvard Rodent Ventilator, Harvard) [23]. The body temperature was maintained at 37 °C with a heating pad. After left lateral thoracotomy, the LAD was occluded for 20 min with an 8-0 nylon suture with a piece of gauze placed between suture and heart to prevent arterial injury. To achieve a 1 µM final lycopene concentration in blood plasma, 4 µL of 15.6 µM lycopene in 0.1% DMSO or 4 µL of 0.1% DMSO per gram body weight were administered via tail vein injection immediately following 20 min of LAD ligation. ECGs were monitored to confirm the ischemic hallmark of ST-segment elevation during coronary occlusion (ADInstruments, Colorado Springs, CO, USA). Immediately following reperfusion, the left ventricle was separated before freeze clamp in liquid nitrogen and stored in −80 °C for further use.

### 2.4. Measurement of Myocardial Infarct

Infarct size was measured as previously described [24]. In short, hearts were reperfused for 4 h then dual stained. Non-necrosis tissue in the ischemic region was stained red by TTC (1%, *w*/*v*) and the non-ischemic region was stained blue with Evan’s blue (1%, *w*/*v*). Hearts were fixed in 4% formalin overnight at 4 °C and then sectioned into 1 mm slices in a matrix, photographed using a Leica microscope and analyzed by the use of Image J 1.49 software for Mac OS X (available at http://rsb.info.nih.gov/ij/). The myocardial infarct size was calculated as the extent of myocardial necrosis to the percentage of ischemic area at risk (AAR).

### 2.5. MDA Assay

Cardiac tissue malondialdehyde (MDA) contents were measured using a Lipid Peroxidation (MDA) Assay Kit (Abcam, Cambridge, UK) according to manufacturer’s instruction. Briefly, after 20 min of LAD ligation followed by 20 min of reperfusion, left ventricles were immediately freeze clamped in liquid nitrogen then stored in an −80 °C freezer. 10 mg of each tissue sample was homogenized on ice in 300 µL of MDA lysis buffer (with 3 µL 100X BHT) then centrifuged at 13,000 g for 10 min. Two hundred microliters of supernatant from each sample was collected and combined with 600 µL of TBA solution. Then, 2 mM MDA stock solution was diluted in ddH_2_O to generate 200 µL 0, 4, 8, 12, 16 and 20 nmol standards, which were then developed with 600 µL of TBA solution. The development of samples and standards was incubated at 95 °C for 60 min and then allowed to cool on ice for 10 min. Technical replicates of each sample consisted of 200 µL of each and absorbance was measured at 532 nm.

### 2.6. Immunoblotting Analysis

Western blots were performed as previously described [25,26]. In brief, heart tissue samples were homogenized in ice-cold lysis buffer and protein concentration was determined using the Bradford method (Bio-Rad Laboratories, Hercules, CA, USA). Heart homogenate proteins were then resolved by SDS-PAGE and transferred onto polyvinylidene difluoride (PVDF) membranes. For reprobing, membranes were stripped with Restore Western Blot Stripping Buffer from Thermo Scientific (Rockford, IL, USA). Rabbit polyclonal antibodies against AMPK, phosphor-AMPK (Thr^172^), ACC, phosphor-ACC (Ser^79^), p44/42 MAPK, phosphor-p44/42 MAPK (Thr^202^/Tyr^204^), p38 MAPK, phosphor-p38 MAPK (Thr^180^/Tyr^182^), SAPK/JNK, phosphor-SAPK/JNK (Thr^183^/Tyr^185^) and horseradish peroxidase-linked secondary antibody were purchased from Cell Signaling (Danvers, MA, USA).

### 2.7. Statistical Analysis

Data are expressed as mean ± SEM, and significance is tested by Student’s unpaired two-tail *t* tests or two-way repeated measures ANOVA with Bonferroni correction with *p* < 0.05 considered significant.

## 3. Results

### 3.1. Lycopene Protects against I/R Induced Cardiomyocyte Death

**Figure 2:**
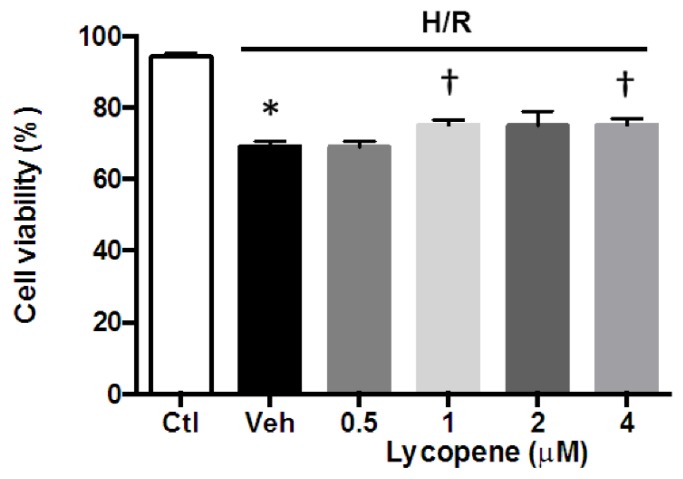
Effect of lycopene on HL-1 cardiomyocytes viability during *in vitro* hypoxia/reoxygenation (H/R). HL-1 cells were subjected to 2 h of hypoxia, then treated with 0.5, 1, 2, 4 μM lycopene or vehicle alone during 2 h of reoxygenation. Controls were exposed normaxia throughout the experiment. Cell viability was assessed by trypan blue exclusion. Values are means ± SEM from three independent experiments, * *p* < 0.01 *vs.* Ctl, † *p* < 0.01 *vs.* Veh.

To determine whether lycopene is effective in protecting cardiomyocytes against I/R induced cell death and the appropriate dosage for *in vivo* experiments, we tested the viability of HL-1 cardiomyocytes in an *in vitro* H/R model by exposing cells to 2 h of hypoxia followed by 2 h of reoxygenation as well as varying concentrations of lycopene. Results show that hypoxia/reoxygenation (H/R) alone significantly reduced cell viability when compared to control (69.14% ± 0.79% *vs.* 94.18% ± 0.50%, *p* < 0.001) (Figure 2). Cells treated with 0.5 μM lycopene did not attenuate cell death (69.00% ± 0.98%). However, higher dosages of lycopene (1 μM, 2 μM and 4 μM) resulted in 75.16% ± 0.76%, 74.97% ± 2.36%, and 75.13% ± 1.05% cell viability, respectively, demonstrating a significant improvement of cell survival compared to the vehicle group. However, dosages over 1 μM failed to achieve higher cell viability than 1 μM lycopene treatment. These data suggest that lycopene is protective against H/R induced cardiomyocyte death *in vitro*.

### 3.2. Effects of Intravenous Administration of Lycopene on I/R Induced Myocardial Infarct

**Figure 3:**
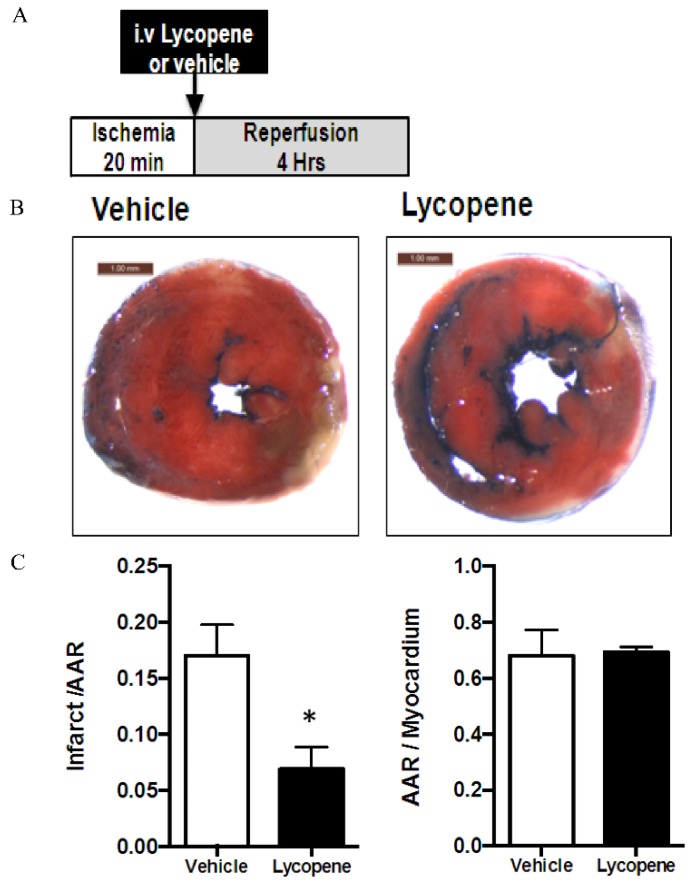
Lycopene (Lyc) reduces myocardial infarct size after ischemia-reperfusion (I/R). *In vivo* hearts were subjected to 20 min of ischemia followed by 4 h of reperfusion. Four microliters of 15.6 μM lycopene (in 0.1% DMSO saline solution, *v*/*v*) or vehicle per gram bodyweight was administrated via tail vein injection after 20 min of LAD ligation. The extent of myocardial necrosis was assessed by TTC staining. (**A**) A scheme showing experiment design; (**B**) representative sections of the extent of myocardial infarction; and (**C**) ratio of the infarct size to the area at risk (AAR) (**left**) and the ratio of AAR to the total myocardial area (**right**). Values are means ± SEM of ≥4 in each group, * *p* < 0.01 *vs.* vehicle.

To confirm the cardioprotective effects of lycopene observed in the *in vitro* H/R cell model, we next examined whether acute lycopene treatment was able to attenuate myocardial infarction in response to *in vivo* regional I/R. After 20 min of left descending coronary artery ligation [27], lycopene was administered to animals through tail vein injection followed by 4 h of reperfusion (Figure 3A). Lycopene administration remarkably decreased the myocardial infarct size compare to the vehicle group (6.89% ± 0.99% of AAR *vs.* 17.06% ± 1.12% of AAR, *n* ≥ 4 in each group, *p* < 0.01) (Figure 3B,C). While there is no difference in AAR to myocardium ratio between lycopene treatment and vehicle group (69.42% ± 0.92% *vs.* 68.21% ± 3.71%) (Figure 3D). These data strongly suggest that acute intravenous administration of lycopene ameliorates I/R-induced myocardial injury in mice.

### 3.3. Effect of Lycopene on ROS Generation during I/R

**Figure 4:**
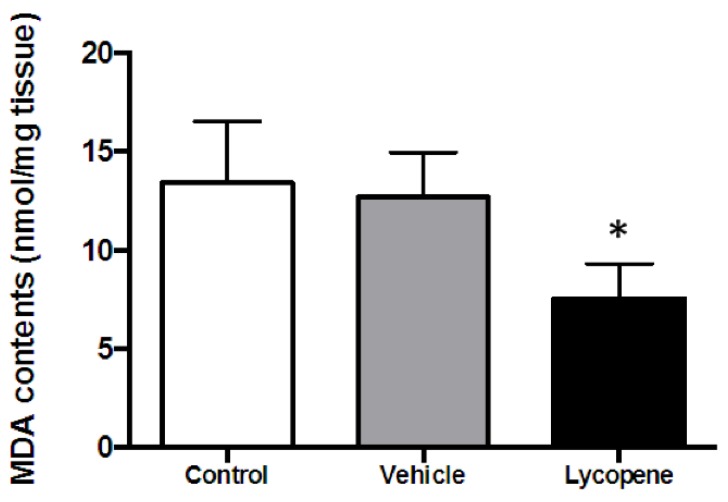
Malondialdehyde (MDA) levels of *in vivo* I/R cardiac tissue. Lycopene or vehicle was given to mice through tail vein injection after 20 min of *in vivo* regional ischemia, then followed by 20 min of reperfusion (I/R); control was not treated with vehicle or lycopene. LVs were harvested immediately after reperfusion then subjected to MDA assay. Values are means ± SEM, *n* ≥ 3 in each group, * *p* < 0.05 *vs.* control and vehicle.

To ascertain whether lycopene reduces MI during I/R by scavenging ROS, we then measured malondialdehyde (MDA) levels in cardiac tissue of the *in vivo* I/R model. Lycopene or vehicle was given through tail vein injection following 20 min of ischemia. After 20 min of reperfusion, cardiac tissue was collected for MDA assay. The data shows that, after 20 min of ischemia followed by 20 min of reperfusion, MDA level in vehicle group is 12.70 ± 1.30 nmol/g tissue, which has no difference with the control group (13.41 ± 1.80 nmol/g) (Figure 4). However, the lycopene treatment group exhibited a significant reduction in MDA production (7.54 ± 1.02 nmol/g) compared to both control and vehicle groups (*n* ≥ 3 in each group, *p* < 0.05), showing that lycopene reduced ROS generation during acute myocardial I/R. These results indicate the cardioprotective effect against infarction of lycopene may associate with its antioxidant properties.

### 3.4. Signaling Pathways Regulated by Acute Lycopene Treatment during I/R

**Figure 5:**
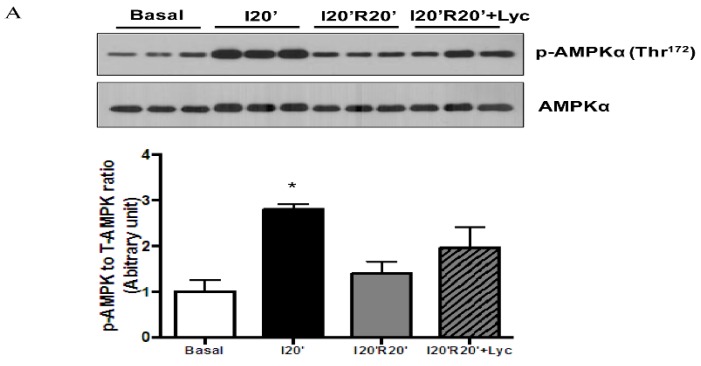

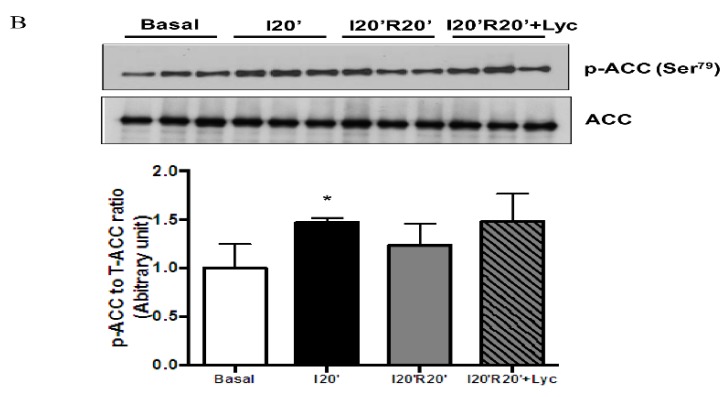
Effects of lycopene (Lyc) on fatty acid oxidation regulating signaling during *in vivo* I/R: (**A**) lycopene treatment moderately elevated AMPK phosphorylation at Thr^172^ during reperfusion, * *p* < 0.01 *vs.* basal; and (**B**) lycopene mildly augmented ACC phosphorylation at Ser^79^ during reperfusion, * *p* < 0.01 *vs.* basal. Values are means ± SEM, *n* ≥ 3 in each group.

Fatty acid β-oxidation is the major source of ROS generation during myocardial I/R. To further determine whether reduced MDA production by lycopene is derived from suppressing fatty acid β-oxidation, we measured a key regulator of fatty acid metabolism, AMP activated kinase (AMPK), and its direct downstream effector, acetyl-CoA carboxylase (ACC) [28], a rate-limiting enzyme for fatty acid β-oxidation in our *in vivo* myocardial I/R mouse model. Western blotting data illustrated that 20 min of ischemia resulted in a significant increase in AMPK phosphorylation (Figure 5A) while 20 min of reperfusion reduced AMPK phosphorylation levels similar to those at baseline. We also observed a moderate upregulation of AMPK following lycopene treatment (Figure 5A). Phosphorylation of ACC was significantly elevated by ischemic stress followed by dephosphorylation during reperfusion. However, in the presence of lycopene, ACC remained phosphorylated during reperfusion (Figure 5B). This suggests that lycopene does not inhibit fatty acid oxidation during myocardial I/R rather it moderately activates the AMPK-ACC signaling pathway. Therefore, the reduction of cardiac MDA level during I/R by lycopene administration may be a direct result of its antioxidative properties.

**Figure 6:**
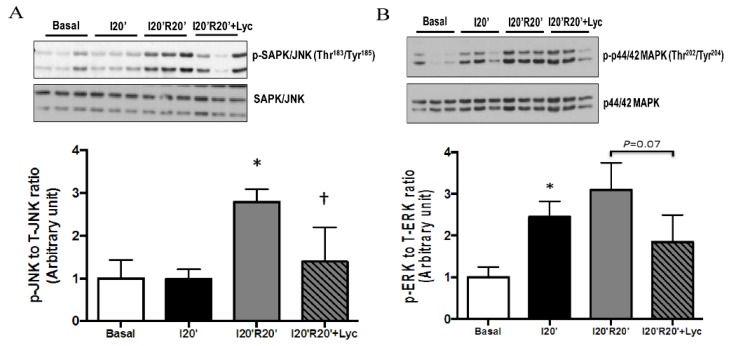

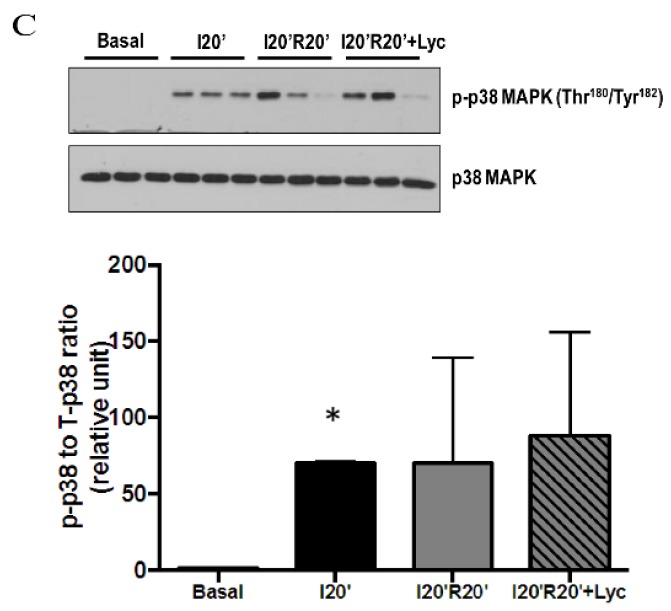
Effects of acute lycopene (Lyc) administration on MAPK signaling pathways during *in vivo* I/R: (**A**) lycopene administration significantly attenuated JNK phosphorylation at Thr^183^/Tyr^185^ during reperfusion, * *p* < 0.01 *vs.* basal and I20′, † *p* < 0.05 *vs.* I20′R20′ vehicle; (**B**) lycopene reduced p42/44 phosphorylation at Thr^202^/Tyr^204^ during reperfusion, * *p* < 0.01 *vs.* basal, *p* = 0.07 *vs.* I20′R20′ vehicle; and (**C**) lycopene has no impact on p38 phosphorylation during reperfusion, * *p* < 0.01 *vs.* basal. Values are means ± SEM, *n* ≥ 3 in each group.

Mitogen-activated protein kinases (MAPK) are a protein family of serine-threonine kinases that play a critical role in numerous cellular processes in response to environmental stress as well as regulating inflammation [29]. To further explore whether lycopene has an impact on modulating MAPK signaling pathways and inflammation during I/R, we then examined the total expression levels and phosphorylation levels of JNK, p44/42 MAPK and p38 MAPK in cardiac tissue from the *in vivo* I/R model. Results showed that phosphor-JNK (Thr^183^/Tyr^185^) was dramatically elevated by I/R, but significantly downregulated by lycopene treatment, in the absence of change in total protein (Figure 6A). Similarly, phosphor-p44/42 MAPK (Thr^202^/Tyr^204^) to total p44/42 MAPK ratio revealed a trend of reduction by lycopene administration compared to vehicle treatment during reperfusion (Figure 6B), while we did not observe significant effects of acute lycopene treatment on p38 MAPK phosphorylation in our study due to large variation in experimental groups (Figure 6C). Taken together, acute lycopene administration via IV injection ameliorates inflammation by attenuating the JNK signaling pathway during I/R.

## 4. Discussion

Lycopene has been shown to be beneficial for I/R injury in a variety of organs including brain [30], testicle [31], and heart [17,18], suggesting it could be clinically utilized for IHD management. However, the *in vivo* distribution pattern of lycopene determined by the use of isotope and mass spectrometry demonstrated that it is predominantly deposited to liver after digestion [19,20]. Moreover, it has been reported that no further increases in serum or tissue concentrations in rats fed with dietary lycopene over 0.5 g/kg/day [32], suggesting there is a strong first-pass metabolism for lycopene and thereby the relatively low bioavailability restricts the therapeutic application of lycopene through oral intake. Thus, the effectiveness of oral uptake of lycopene for treating ischemic diseases has been questioned. On the contrary, intravenous administration can rapidly and significantly increase circulating lycopene levels and could be more efficient at protecting against myocardiac I/R injury.

In the present study, we firstly examined whether acute lycopene administration is able to prevent I/R induced cardiomyocyte death. Our data indicates that acute treatment of 1 μM lycopene is effective to improve cardiomyocyte viability during *in vitro* H/R, which is consistent with previous findings from H9C2 fibroblasts [33]. These findings suggest that extracellular lycopene concentration may be critical for preventing ROS induced cell death during I/R. In addition, we also determined that lycopene concentrations higher than 1 μM did not further improve cell viability, which is consistent with recent report from Gao *et al.*, which demonstrated that 1.25 μM lycopene treatment significantly elevated the survival rate of H9C2 cells during H/R, while increase the dosage to 20 μM did not achieve further improvement [34].

Yue *et al.* have shown that lycopene-fed rats have smaller infarct size after cardiac I/R [35], to investigate whether IV administration of lycopene could also protect against I/R induced MI, we then established an *in vivo* myocardial I/R mouse model. Since our cell experiments have demonstrated 1 μM lycopene is effective to suppress H/R induced cardiomyocyte death, and Schierle *et al.* reported that lycopene concentrations in human blood plasma range from 0.2 to 1 μM [28], we sought to determine whether 1 μM of lycopene prevents I/R induced MI *in vivo*. To achieve a 1 μM lycopene in blood circulation, 4 µL of 15.6 µM lycopene in 0.1% DMSO per gram body weight given to mice through tail vein injection immediately before reperfusion. TTC dual staining results suggest that 1 μM lycopene is sufficient to attenuated I/R induced MI in mice. To the best of our knowledge, we are the first group to report that IV lycopene administration alleviates I/R induced MI, and our evidence strongly suggests that IV lycopene administration could be a potential therapeutic approach for I/R injury management.

It is known that myocardial ischemia breaks the homeostasis between energetic substrate delivery within the blood stream and the metabolic requirements of the heart [36]. Hence, during ischemia, the activity of mitochondria is inhibited due to oxygen shortages [37], which in turn leads to cardiomyocytes undergoing nutrient deprivation and stress. As a consequence, reperfusion after a short ischemic episode promotes oxidative phosphorylation to compensate for the energy loss. Meanwhile, ROS is dramatically generated from ETC [2], which will further impair mitochondrial respiratory function [38], exacerbate cardiomyocyte contractile malfunction and cause other symptoms of cardiomyopathy [39]. It has been reported that lycopene attenuates H_2_O_2_ induced apoptosis in H9C2 cell [35], which implies the cardioprotective effect of lycopene is associated with ROS scavenging. To study whether IV lycopene administration protects against cardiac I/R injury by reducing ROS generation during reperfusion, we next measured MDA levels in our *in vivo* myocardial I/R model. Our results demonstrate that acute lycopene administration effectively reduced MDA production during reperfusion indicating that cardiac ROS accumulation during I/R is suppressed by lycopene treatment. This finding is consistent with multiple reports that show lycopene decreases ROS levels in varies models [35,40]. Although previous studies also suggest that lycopene upregulates antioxidant response elements and nuclear factor E2-related factor 2 [41], increases of antioxidant enzymes expression including SOD, CAT, the metal binding protein transferrin, and epoxide hydrolase 1 [42], the effect of lycopene on antioxidants expression may not be the primary protective mechanism in the setting of short-time I/R induced myocardial injury.

To further distinguish whether the cardioprotective effects of lycopene are derived from its direct antioxidative properties or its effects on modulating fatty acid oxidation, we next analyzed the AMPK-ACC signaling pathway in the *in vivo* I/R model. Our data show that acute lycopene moderately promoted AMPK-ACC signaling pathway activation during reperfusion. Although the observed changes were not statistically significant, it indicates that capturing ROS rather than inhibiting fatty acid utilization contributes to lower ROS accumulation during I/R by acute lycopene administration. Interestingly, it is known that AMPK exhibits cardioprotective effects during I/R injury by promoting GLUT4 mediated glucose uptake [43]. Santulli *et al.* have reported that abnormal Ca^2+^ release from sarcoplasmic reticulum is a key step for postischemic heart failure [44], giving that calmodulin-dependent kinase kinase beta (CaMKKβ) is an upstream regulator of AMPK [45], it will be interesting to investigate whether lycopene could regulate Ca^2+^ signal and mitochondrial function in cardiomyocyte during I/R. Moreover, to further explore the correlation between lycopene and AMPK during cardiac I/R, the use of an AMPK knockout animal model will be very helpful to better understand the mechanism of lycopene in attenuating I/R induced MI.

Previous studies have demonstrated that MAPK can be activated by stress and inflammatory stimuli [29] while several studies have documented that myocardial I/R is associated with MAPK activation [46,47], and pharmacological inhibition of JNK is beneficial for limiting I/R injury [48,49] We report that along with reduced myocardial infarct size, acute lycopene administration also significantly inhibited JNK phosphorylation at Thr^183^/Tyr^185^ during reperfusion, which is consistent with previous findings that lycopene treatment inhibits H_2_O_2_ induced JNK phosphorylation [50]. Furthermore, we report that lycopene treatment lowers p44/42 MAPK phosphorylation during reperfusion compare to vehicle. Previous studies have shown that p44/42 MAPK phosphorylation during I/R is cardioprotective [47,51]. However, in these studies, pharmacological p44/42 MAPK inhibitors, PD98059 and UO126 were utilized to examine the function of p44/42 MAPK during cardiac I/R, both of which dramatically inhibited p44/42 MAPK activation and resulted in worsening I/R injury. In our study, p44/42 MAPK phosphorylation in lycopene treated groups was found to be lower than vehicle groups during reperfusion but still maintained a notable increase compared to the basal condition. Reduction of p44/42 MAPK phosphorylation by lycopene may result from less ROS stress. Although p38 MAPK activation is also associated with ischemic stress [52,53], we report no difference in p38 MAPK phosphorylation during I/R as a result of lycopene administration.

In summary, lycopene is a natural potent antioxidant and has long been associated with human health. We utilized both *in vitro* and *in vivo* I/R models to evaluate the effects of lycopene on cardiomyocyte following I/R injury. In this study, we first report that acute lycopene administration through IV injection protects against I/R induced MI. In addition, our data also elucidated that JNK activation during reperfusion is suppressed by acute lycopene administration, which indicating lycopene ameliorate inflammation during I/R probably due to its effects on scavenging ROS. Nevertheless, other groups have suggested that lycopene reduces myocardial I/R injury by promoting autophage, and alleviating endoplasmic reticulum stress [54,55]. Taken together, these findings indicate that lycopene may prevent myocardial I/R injury through multiple pathways and mechanisms. There are many limitations still remain in this study, such as the dose–response curve of lycopene for *in vivo* I/R is missing, did not provide cardiac troponin I value to assess MI; and failed to perform postischemic cardiac contractile function measurement. In addition, we acknowledge the working concentration of lycopene in our study is too high to be used clinically. Therefore, we feel that further studies on more potent lycopene isomers and/or derivatives need to be conducted due to lycopene’s potential therapeutic effects in treating IHD.

## 5. Conclusions

In conclusion, the present study reveals that 1 μM lycopene treatment immediately before reoxygenation significantly represses H/R induced cardiomyocytes death. Moreover, supplement of 1 μM circulating lycopene in blood via IV injection effectively reduced MI during *in vivo* I/R in mice, and significantly inhibited fatty acid oxidation and JNK signaling activation during reperfusion.
